# Knockout of the ABCB1 Gene Increases Susceptibility to Emamectin Benzoate, Beta-Cypermethrin and Chlorantraniliprole in *Spodoptera frugiperda*

**DOI:** 10.3390/insects13020137

**Published:** 2022-01-27

**Authors:** Qi Li, Minghui Jin, Songmiao Yu, Ying Cheng, Yinxue Shan, Peng Wang, Haibin Yuan, Yutao Xiao

**Affiliations:** 1 College of Plant Protection, Jilin Agricultural University, Changchun 130118, China; liqi9150@163.com; 2 Shenzhen Branch, Guangdong Laboratory of Lingnan Modern Agriculture, Genome Analysis Laboratory of the Ministry of Agricultural and Rural Affairs, Agricultural Genomics Institute at Shenzhen, Chinese Academy of Agricultural Sciences, Shenzhen 518120, China; jinminghui@caas.cn (M.J.); ysm18845726086@163.com (S.Y.); canoecheng@163.com (Y.C.); shanyinxue9@163.com (Y.S.); wpeng20@163.com (P.W.)

**Keywords:** *Spodoptera frugiperda*, ABCB1, CRISPR/Cas9, insecticides, Bt toxins

## Abstract

**Simple Summary:**

*Spodoptera frugiperda* is an invasive lepidopteran pest that causes extensive damage to many commercial crops. To control this pest, the use of chemical pesticides has become increasingly important because of their high efficiency, low cost and ease of mechanized operations. However, despite the proven effectiveness of pesticides, the long-term use of chemicals not only pollutes the environment, but also leads to resistance in field populations, making pest control more difficult. In this study, we used the CRISPR/Cas9 system to generate an ABCB1 knockout strain to explore its potential role in determining susceptibility to chemical pesticides or Bt toxins. We compared the mortality of WT strains and knockout strains treated with 10 insecticides and three Bt proteins, and found that the insects were significantly more susceptible to the three insecticides (emamectin benzoate, beta-cypermethrin and chlorantraniliprole) after ABCB1 was knocked out. Our results showed that ABCB1 is closely related to the detoxification metabolism of some pesticides, and suggest that the resistance of *Spodoptera frugiperda* to emamectin benzoate, beta-cypermethrin and chlorantraniliprole may be related to the overexpression of ABCB1. This lays a foundation for a follow-up study on the resistance mechanism of *Spodoptera frugiperda*.

**Abstract:**

ATP-binding cassette transporter B1 (ABCB1, or P-glycoprotein) is known to be an important participant in multidrug resistance in mammals, and it also has been proved as a transporter for some insecticides in several lepidopteran insects, yet the precise function of this transporter in *Spodoptera frugiperda* is unknown. Here, we generated a *SfABCB1* knockout strain of the *S. frugiperda* using the CRISPR/Cas9 system to explore its potential roles in determining susceptibility to chemical insecticides or Bt toxins. Bioassay results showed that the susceptibility of *SfABCB1* knockout strain to beta-cypermethrin, chlorantraniliprole and emamectin benzoate were significantly increased compared with the wild-type strain DH19, whereas there were no changes to Bt toxins for Cry1Ab, Cry1Fa and Vip3Aa. Our results revealed that *SfABCB1* plays important roles in the susceptibility of *S. frugiperda* to beta-cypermethrin, chlorantraniliprole and emamectin benzoate, and imply that overexpression of ABCB1 may contribute to beta-cypermethrin, chlorantraniliprole and emamectin benzoate resistance in *S. frugiperda*.

## 1. Introduction

The fall armyworm (*Spodoptera frugiperda*, Lepidoptera Noctuidae) is a major polyphagous agricultural pest. *S. frugiperda* is a native pest in tropical and subtropical America, with strong migratory ability, high reproductive capacity, and extremely wide host range, causing serious economic losses [1,2]. In 2018, *S. frugiperda* invaded Yunnan Province in China and spread its damage rapidly [3]. At present, the use of chemical pesticides is the main method to control *S. frugiperda*. Therefore, the problem of field populations’ pesticide resistance needs to be paid great attention.

ATP-binding cassette (ABC) transporters belong to a large subfamily of transmembrane proteins with important physiological functions that are conserved in archaea, eubacteria and eukaryotes [4]. According to the sequence similarity of ATP binding sites, ABC transporters can be divided into eight subfamilies A~H [5,6]. The first ABC transporter (ABCB1, or P-glycoprotein) in a eukaryote was found in humans (*Homo sapiens*) [7], and it is also the first protein found in humans that is resistant to drugs treating cancer. With the rapid increase in the completion of arthropod whole-genome sequencing, research on the the ABC transporter gene family is also growing rapidly. Based on sequence similarity, the ABCB subfamily of insects is considered to be involved in resistance to insecticides and other chemicals [8]. Recent studies have shown that ABC transporters can actively export bound toxins outside the cell and cause xenobiotic resistance [9,10]. In addition, members of the ABC transporter subfamily, such as C2 [11,12,13], A2 [14] and B1 [15], have been shown to play a decisive role in the sensitivity to *Bacillus thuringiensis* (Bt) toxins. However, the role of *SfABCB1* in handling insecticides and Bt toxins is unknown.

In this study, a *SfABCB1* knockout strain of *S. frugiperda* was generated by the CRISPR/Cas9 genome editing system to investigate the relevance of susceptibility of *SfABCB1* to insecticides and Bt toxins. Results showed that the *SfABCB1* knockout strain was more sensitive to beta-cypermethrin, chlorantraniliprole and emamectin benzoate than the wild-type (WT) strain. Our results showed the importance of *SfABCB1* in *S. frugiperda* combating beta-cypermethrin, chlorantraniliprole and emamectin benzoate, and provided further evidence for the participation of the ABCB1 gene in insecticide resistance.

## 2. Materials and Methods

### 2.1. Insects

*S. frugiperda* strain DH19 was collected from Ruili, Yunnan Province of China, in January 2019 [13] and reared in the laboratory on an artificial diet without exposure to any pesticides or Bt toxins. Insects were kept at 27 ± 2 °C and 75 ± 10% relative humidity (RH) with 14 h light:10 h dark photoperiod. Adults were provided 10% sucrose solution for nutrition.

### 2.2. Insecticides and Bt Toxins

Formulated insecticides (emamectin benzoate, beta-cypermethrin, chlorantraniliprole, indoxacarb, tebufenozide, bifenthrin, chlorpyrifos, abamectin, chlorfenapyr and decamethrin) used in bioassays were provided by Prof. Chaobin Xue (Shandong Agricultural University, Tai’an, China). The Cry1Ab, Cry1F and Vip3Aa toxins used in this study were obtained from the Institute of Plant Protection, Chinese Academy of Agricultural Sciences (CAAS) (Beijing, China).

### 2.3. Design and Preparation of sgRNA

**Figure 1:**
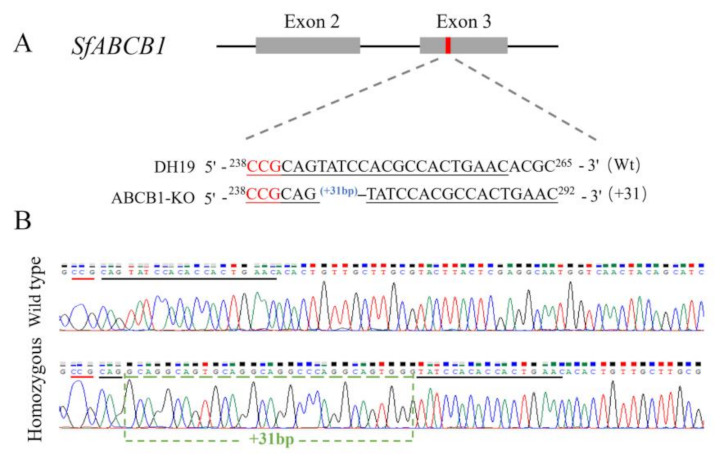
Schematic diagram of the knockout of *SfABCB1*. (**A**) Schematic diagram of the sgRNA-targeting sites. The black line indicates the genome locus of *Spodoptera frugiperda* ABCB1 (*SfABCB1*) and the two grey boxes represent the two exons of *SfABCB1*. The sgRNA-targeting site was located on the sense strand of exon-3. The sgRNA-targeting sequence is in black, and the protospacer adjacent motif (PAM) sequence is in red. (**B**) Representative chromatograms of polymerase chain reaction (PCR) product sequencing of G0 moths, and the indel mutation is presented in the green box.

The small guide RNA (sgRNA) for editing the *SfABCB1* gene was designed using the sgRNAcas9 design tool [16]. After on-target and off-target analysis, a sgRNA-ABCB1 target sequence (5′-CCGCAGTATCCACGCCACTGAACACGC-3′) was selected at exon2 (Figure 1A). The selected sgRNA was checked by comparing it against the *S. frugiperda* genome (https://bipaa.genouest.org) (accessed on 1 March 2021) for potential off-target sites. The template DNA for in vitro transcription of the sgRNA was made using PCR-based fusion of two oligonucleotides with the T7 promoter. In vitro transcription was performed with the GeneArt Precision gRNA Synthesis Kit (Thermo Fisher Scientific, Waltham, MA, USA), according to the manufacturer’s instructions. Cas9 protein was purchased from Thermo Fisher Scientific, USA.

### 2.4. Egg Collection and Microinjection

Eggs freshly laid (within 2 h after oviposition) were washed with distilled water and then placed on a glass slide and fixed with double-sided adhesive tape. About 1 nL mixture of sgRNA (150 ng/μL) and Cas9 protein (50 ng/μL) was injected into individual eggs using a microinjector (Nanoject III, Drummond, Broomall, PA, USA). After injection, eggs were incubated at 25 °C and 65% RH for hatching.

### 2.5. Analysis of CRISPR/Cas9-Induced Mutations of SfABCB1

For analysis of mutations, PCR fragments flanking the targeted sites were amplified with ABCB1 primers (SFB1-E3-4F: AGGGACAAACTCTTCATAACC, SFB1-E3-4R: TGGCAGCGTAAGACAGAA). The genomic DNA was extracted with a Multisource Genomic DNA Miniprep Kit (Axygen, New York, NY, USA) according to the manufacturer’s instructions. The PCR conditions were: 94 °C for 5 min, 34 cycles of 94 °C for 30 s, 60 °C for 30 s, 72 °C for 35 s, followed by a final extension period of 72 °C for 10min. PCR amplicons were then analyzed via agarose gel electrophoresis. Direct sequencing of PCR products was conducted and double sequencing peaks, indicating a mutation event, were identified. PCR products were then recovered and cloned into pEASY-T3 Vector (TransGen, Beijing, China) and sequenced by Sangon Biotech (Shanghai, China).

### 2.6. Insect Bioassays

The toxicity of each pesticide and Bt toxin to DH19 and the *SfABCB1* knockout strain was measured by diet overlay bioassays. A volume of 900 μL warm artificial diet was dispensed into a 24-well plate (surface area = 2 cm^2^). The formulated insecticides were diluted to generate five serial solutions with dimethyl sulfoxide (DMSO). For Cry1Ab, Vip3Aa and Cry1Fa toxins, gradient concentrations of toxin solutions were prepared with distilled water. Control groups were treated with DMSO or distilled water, respectively. After the diet cooled down, a volume of 40 μL of the solutions above was applied on the diet surface in each well and left to dry.

For the chemical insecticide treatments, a one third-instar larva was placed in each well, and 24 larvae were used for each concentration. Mortality was scored after 2 days. Larvae were considered dead if they did not move with gentle prodding.

For Cry1Fa, Cry1Ab and Vip3Aa toxin treatments, newly hatched neonates were 1 larva in each well tested and mortality was recorded after 7 days. For all three toxins, larvae were considered dead if they did not move or weighed less than 3 mg at the end of bioassays.

The LC_50_ values (the concentration of pesticide or Bt protein that will kill 50% of larvae) and the corresponding 95% fiducial limits were calculated through probit analysis of the mortality data using DPS. LC_50_ values were considered significantly different if fiducial limits did not overlap.

## 3. Results

### 3.1. CRISPR/Cas9 Mediates Efficient SfABCB1 Knockouts

A mixture of Cas9 protein and in vitro transcribed sgRNA were co-injected into 200 newly laid eggs to target ABCB1 in *S. frugiperda*. After injection, 18.5% (37/200) of injected eggs hatched and 78.4% (29/37) of the neonates developed into adults (G0). Male and female moths of G0 were backcrossed with the strain DH19 on a large scale, and the next generation (G1) was produced by single pair mating. After sufficient eggs were collected, genomic DNA samples of individual G0 moths were prepared, and PCR products were directly sequenced to screen *SfABCB1* genotypes. The results indicated that 20.1% (6/29) of the G0 moths were mutagenized. PCR products of the target sequence were TA-cloned and sequenced to determine the exact mutations.

### 3.2. Mutation Inheritance

**Figure 2:**
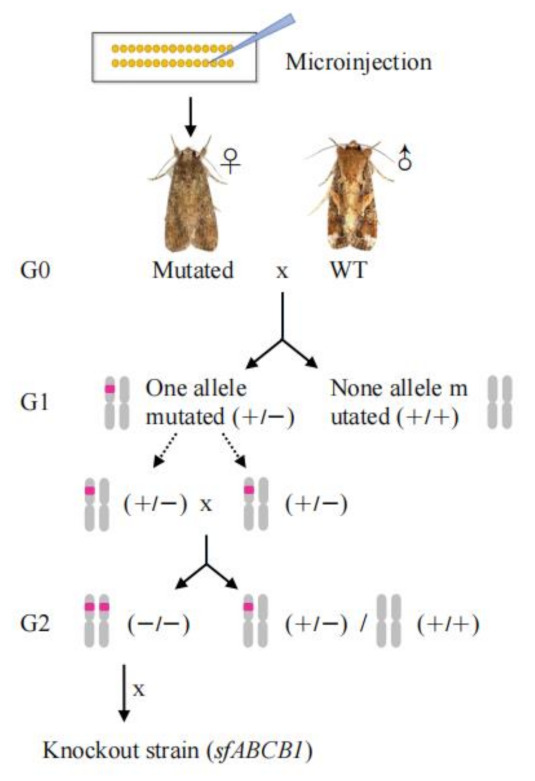
Diagram detailing crossing strategy to obtain a homozygous strain. The G0 moths were heterozygous mutant after microinjectionDH19. Moths from DH19 and mutant G0 moth were mated to obtain G1 generation. Heterozygous G1 individuals were mass-crossed to obtain G2 generation, and 14.6% (14/96) of the tested individuals were identified as homozygous. These homozygous individuals were pooled to produce a *SfABCB1* knockout strain (ABCB1-KO).

To generate a homozygous knockout strain, a strain with 31-bp insertion among the detected mutations was selected (Figure 1B). G1 larvae obtained from the hybridization of 31-bp insertion G0 moth and DH19 stain were reared to pupae, and 23 out of 96 pupae carrying the 31-bp insertion allele were identified by testing exuviates of final instar larvae. Adults from these pupae were mass-crossed to obtain a G2 generation. Ninety-six individuals from the G2 were genotyped using final instar exuviates, and 14.6% (14/96) were identified as homozygous. These homozygous individuals were pooled to produce a *SfABCB1* knockout strain (ABCB1-KO) (Figure 2).

### 3.3. Effect of ABCB1 Knockout on Susceptibility of S. frugiperda to Insecticides

Larvae of the homozygous knockout strain were bioassayed to determine their susceptibility to insecticides relative to the unedited strain. The susceptibility of third instar larvae to emamectin benzoate, beta-cypermethrin, and chlorantraniliprole was significantly increased (based on non-overlapping fiducial limits) by an estimated 4.18-fold, 2.94-fold and 2.89-fold, respectively (based on (LC_50_) values) in the knockout strain ABCB1-KO compared to the WT strain. The bioassay results for chlorantraniliprole also showed a significant increase of an estimated 2.94-fold in susceptibility in *SfABCB1* compared to the WT larvae. There was a 2.89-fold increase in susceptibility to beta-cypermethrin in *SfABCB1* compared to the WT larvae. Results from bioassays with other insecticides showed no significant changes of susceptibility in ABCB1-KO compared to WT larvae (Table 1).

**Figure 3:**
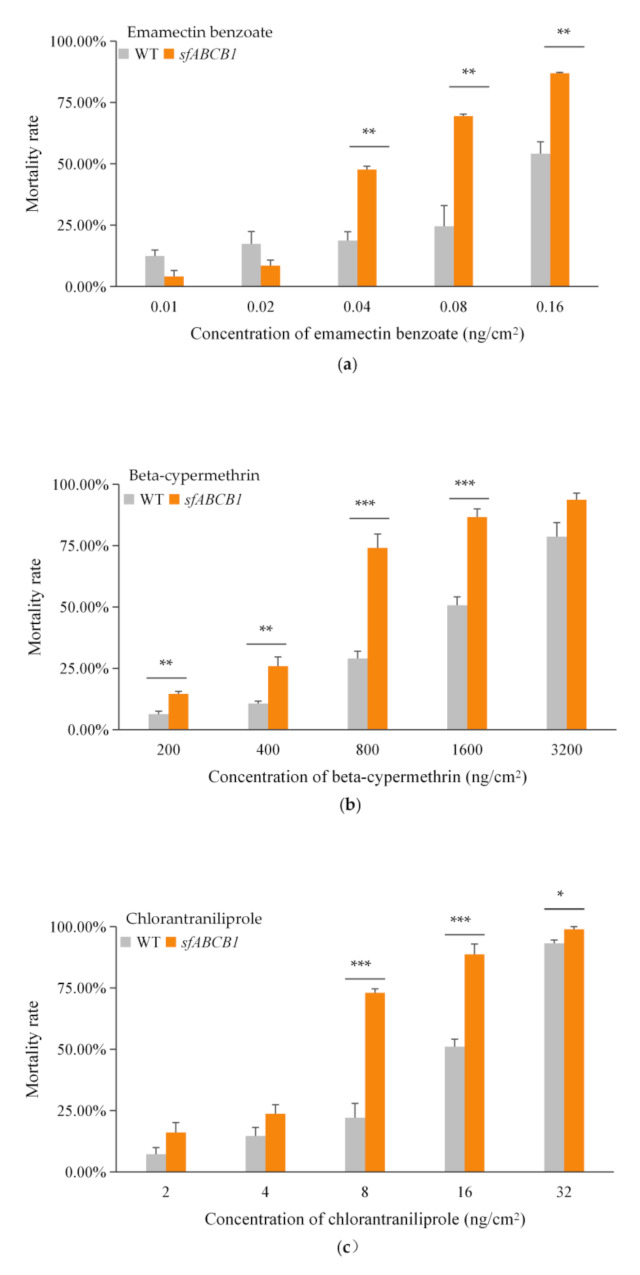
Mortality rate of DH19wild type and *sfABCB1* strains at different concentrations of (**a**) emamectin benzoate; (**b**) beta-cypermethrin and (**c**) chlorantraniliprole. *, *p* < 0.05; **, *p* < 0.01; ***, *p* < 0.001.

The bioassay results showed that the difference in mortality between WT and ABCB1-KO was the largest after treating with emamectin benzoate, followed by beta-cypermethrin and chlorantraniliprole. By comparing the results of different concentrations of each toxin tested, we found that the trends of larvae mortality were not always the same when treated with emamectin benzoate, beta-cypermethrin or chlorantraniliprole. For emamectin benzoate, the mortalities of *sfABCB1* were less than that of WT at low concentrations. However, the mortality ratios of *sfABCB1* were significantly higher than that of WT when treated with emamectin benzoate higher than 0.04 ng/cm^2^ (Figure 3a). For beta-cypermethrin and chlorantraniliprole, the mortality rate of *sfABCB1* strain was always higher than that of WT strain (Figure 3b,c). However, no significant differences were detected between the two strains treated with 3200 ng/cm^2^ beta-cypermethrin, and 2 ng/cm^2^ and 4 ng/cm^2^ chlorantraniliprole only (Figure 3b,c).

### 3.4. Susceptibility of ABCB1 Knockout Neonates to Bt Toxins

To determine the potential roles of *SfABCB1* in the toxicology of Bt toxins, we performed a bioassay using Cry1Ab, Cry1Fa and Vip3Aa. The results showed no significant changes in the susceptibility of *SfABCB1* knockout strain compared with the wild-type strain DH19 when treated with the three Bt toxins (Table 2).

## 4. Discussion

ABC transporters play an indispensable role in multidrug resistance in both eukaryotes and prokaryotes, where ABCB1 (p-glycoprotein) acts as a widely specific cellular efflux pump for hydrophobic organic chemicals in mammals that has been widely studied [18,19]. After the ABC gene was proved to be related to multidrug resistance in humans, the role of the insect ABC gene in insecticide resistance has attracted increasing attention. However, the functional correlation between ABC transporters and drug resistance in arthropods is limited. In this study, the ABCB1 gene was edited by reverse genetics and its functional relevance to susceptibility of pesticides (emamectin benzoate, beta-cypermethrin and chloranthramide) was confirmed.

The CRISPR/Cas9 genome editing system has been successfully used to manipulate different target genes in many non-model species [20,21], including *S. frugiperda* [22]. In this study, we obtained a mutation frequency of 20.1% in G0. As one of the main criteria to evaluate the efficiency of gene editing techniques, mutation frequency may be affected by a variety of factors, such as target gene selection, sgRNA or Cas9 dose, and the form of Cas9 (plasmid, mRNA or Cas9 protein) [23]. Microinjection techniques (injection location, injection depth and needle size) also affect mutation frequency and G0 survival rate [24].

**Figure 4:**
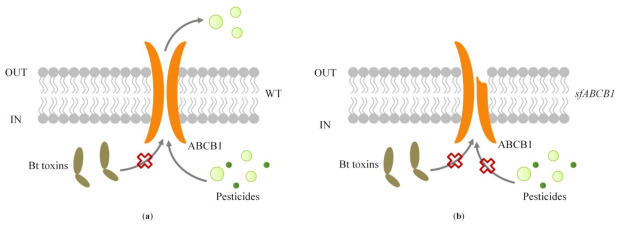
Model to explain the role of ABCB1 in *Spodoptera frugiperda* in increasing susceptibility to emamectin benzoate, beta-cypermethrin, and chlorantraniliprole in vivo. (**a**) Model of ABCB1 in wild type DH19strain (WT). ABCB1 can transport some pesticides extracellular, but not Bt; (**b**) Model of ABCB1 in knockout strain (*sfABCB1*). The deformed ABCB1 protein cannot perform normal transportation functions, resulting in cellular toxins accumulation.

It is reported that ABCB1 is related to resistance to insecticides [6]. Some studies have shown that the expression of ABCB1 in the insecticide-resistant strains is significantly increased. The ABCB1 activity or expression levels in both vertebrates and invertebrates were raised when treated with pyrethroids, cyclodienes or organophosphates (OPs) [25,26,27]. Studies have shown that after treating the *Helicoverpa armigera* with a high concentration of chlorantraniliprole, the expression of ABCB1 increased significantly. Increased levels of ABCB1 protein were also found in populations of *H.armigera* resistant to pyrethroids and OP [26,28]. Knock out of ABCB1 gene in *Spodoptera exigua* resulted in three times the sensitivity to abamectin and emamectin benzoate [29]. In three types of chlorantraniliprole-resistant strains of *Chilo suppressalis*, the expression of ABCB1 was up-regulated in varied degrees [30]. In addition, the synergistic effect of verapamil on pyrethroid toxicity has been confirmed to be correlated with changes in the ABCB1 expression level in *H. armigera* [26,28], while similar findings have been confirmed in *C. pipiens* [25] and *Apis mellifera* [31]. In this experiment, the ABCB1 knockout strain was significantly more susceptible to beta-cypermethrin, chlorantraniliprole and EB. We speculate that the absence of ABCB1 failed to transport beta-cypermethrin, chlorantraniliprole and emamectin benzoate extracellularly, and accumulating toxins in cells raised the sensitivity of *SfABCB1* strain to pesticides (Figure 4).

However, in comparison with emamectin benzoate, beta-cypermethrin, and chlorantraniliprole, knockout of *SfABCB1* showed barely no effect on the sensitivity of *S. frugiperda* to p-indoxacarb, chlorpyrifos, abamectin, tarfenac, tebufenozide, bifenthrin and deltamethrin. Similarly, Bass et al. [25] reported that verapamil had no effect on the toxicity of chlorpyrifos in *C. pipiens*, and no relationship between pyrethroid exposure and ABCB1 expression was detected in *Trichoplusia ni* [32]. Nevertheless, the increase of transcriptional level of ABCB1 can always be detected in the abamectin-resistant strains of *Plutella xylostella* [33]. The knockout of ABCB1 increased the susceptibility of *Spodoptera exigua* to abamectin and emamectin benzoate, but has no effect on the toxicity of chlorantraniliprole and beta-cypermethrin [29]. These results indicate that the defense function of ABCB1 against insecticides varies among different species. Whether this difference is caused by the phenomenon of gene replenishment remains to be further explored. ATP-binding cassette transporters (ABC) include a large family with similar functions and sequences. There are eight ABC transporters in subfamily B. Although they have some differences in functionality, it cannot be ruled out that they have some common functions because they all have common domains. Similar results were reported in Bt receptors, ANP1 and APN6. In a resistance strain of *Trichoplusia ni*, APN1 was significantly down-regulated, whereas APN6 was significantly up-regulated [34].

It has been reported that the down-regulation of *PxABCB1* was associated with Cry1Ac resistance in *P. xylostella* [35]. However, the bioassay results of this study implied that ABCB1 was not the receptor of Cry1Ab, Cry1Fa or Vip3Aa in *S. frugiperda*. Our results are consistent with the *SeABCB1* which was also not involved in the Bt toxins detoxification mode. This difference may be influenced by the different Bt toxins or difference species.

The results of this study provided new evidence for the role of *SfABCB1* in the susceptibility of *S. frugiperda* to emamectin benzoate, beta-cypermethrin, and chlorantraniliprole, and also proved that the CRISPR/Cas9 genome editing system is effective for studying the function of candidate genes in *S. frugiperda*. These results will provide guidance for explaining the insect resistance mechanisms of emamectin benzoate, beta-cypermethrin, and chlorantraniliprole. Due to the short period in which *S. frugiperda* has been invasive in China, no monitoring of its resistance to insecticides has been conducted until now. Therefore, clarifying the resistance mechanism of *S. frugiperda* to pesticides is very important for improving resistance management strategies and the sustainable control of *S. frugiperda*.

## 5. Conclusions

The results of this study provide new functional evidence for *SfABCB1* as a transporter of emamectin benzoate, beta-cypermethrin and chlorantraniliprole, and confirmed that ABCB1 in *S. frugiperda* is not the receptor of the three Bt toxins, Cry1Ab, Cry1Fa and Vip3Aa.

## Figures and Tables

**Table 1:** Susceptibility of third instar larvae from ABCB1 knockout strain (*SfABCB1*) and wild-type strain of *Spodoptera frugiperda* to 10 different pesticides.

Insecticide	Strain	LC_50_ (ng/cm^2^) ^a^	95%FL ^b^	N ^c^	Toxicity Ratio ^d^
Emamectin benzoate	WT	0.1986	0.1392~0.2835	312	4.18 ^e^
	*SfABCB1*	0.0475	0.0389~0.0579	312	
Chlorantraniliprole	WT	15.6798	10.2730~23.9322	240	2.94 ^e^
	*SfABCB1*	5.3417	3.1296~9.1173	240	
Beta-cypermethrin	WT	1263.8372	1069.3669~1493.6731	240	2.89 ^e^
	*SfABCB1*	437.2371	274.4066~696.6900	240	
Indoxacarb	WT	33.4869	27.7322~40.4358	168	2.04
	*SfABCB1*	16.4150	9.0952~29.6256	168	
Tebufenozide	WT	184.2462	95.2849~356.2646	168	1.75
	*SfABCB1*	105.4464	53.0900~209.4357	168	
Bifenthrin	WT	250.9376	189.0816~333.0291	168	1.69
	*SfABCB1*	148.8243	80.1117~276.4723	168	
Chlorpyrifos	WT	1315.3782	880.0563~1966.0331	192	1.42
	*SfABCB1*	924.1850	629.1825~1357.504	192	
Abamectin	WT	2087.3459	1753.1752~2485.2123	192	1.24
	*SfABCB1*	1684.8867	1341.9900~2115.3983	192	
Chlorfenapyr	WT	55.6443	31.3381~98.8027	192	1.20
	*SfABCB1*	46.5237	26.3887~82.022	192	
Decamethrin	WT	94.7553	57.1813~157.0193	192	1.02
	*SfABCB1*	92.5962	58.1709~147.3942	192	

^a^ Lethal concentration that kills 50% of *S. frugiperda* larvae. ^b^ 95% fiducial limits of LC_50_, units are ng toxin per cm^2^ diet. ^c^ Number of larvae tested. ^d^ Toxicity ratio = LC_50_ value for wild-type strain (WT) divided by LC_50_ value for knockout strain (*sfABCB1*). ^e^ LC_50_ of the same toxin significantly greater for the wild-type strain (WT) than *sfABCB1* by the conservation criterion of no overlap of the 95% fiducial limits [17].

**Table 2:** Susceptibility of *Spodoptera frugiperda* neonates from ABCB1 knockout strain (*SfABCB1*) and wild-type strain to 3 Bt toxins.

Insecticide	Strain	LC_50_(μg/cm^2^) ^a^	95%FL ^b^	N ^c^	Toxicity Ratio ^d^
Vip3Aa	WT	19.4096	8.1743~46.0875	192	1.36
	*SfABCB1*	14.2677	4.4004~46.2605	192	
Cry1Ab	WT	14.5213	11.1550~18.9033	264	0.87
	*SfABCB1*	16.6693	13.9074~19.9797	264	
Cry1Fa	WT	0.6803	0.0099~46.5884	192	0.67
	*SfABCB1*	1.0194	0.0231~44.9738	192	

^a^ Lethal concentration that kills 50% of *S. frugiperda* larvae. ^b^ 95% fiducial limits of LC_50_, units are ng toxin per cm^2^ diet. ^c^ Number of larvae tested. ^d^ Toxicity ratio = LC_50_ value for wild-type strain (WT) divided by LC_50_ value for knockout strain (*sfABCB1*).

## Data Availability

No new data were created or analyzed in this study. Data sharing is not applicable to this article.

## References

[1] Nagoshi R.N., Fleischer S., Meagher R.L., Hay-Roe M., Khan A., Murúa M.G., Silvie P., Vergara C., Westbrook J. (2017). Fall Armyworm Migration Across the Lesser Antilles and the Potential for Genetic Exchanges Between North and South American Populations. PLoS ONE.

[2] Gouin A., Bretaudeau A., Nam K., Gimenez S., Aury J., Duvic B., Hilliou F., Durand N., Montagné N., Darboux I. (2017). Two Genomes of Highly Polyphagous Lepidopteran Pests (*Spodoptera Frugiperda*, Noctuidae) with Different Host-Plant Ranges. Sci. Rep..

[3] Li G., Ji T., Sun X., Jiang Y., Wu K., Feng H. (2019). Susceptibility Evaluation of Invaded *Spodoptera Frugiperda* Population in Yunnan Province to Five Bt Proteins. Plant Prot..

[4] Ferro-Luzzi Ames G., Mimura C.S., Holbrook S.R., Shyamala V. (1992). Traffic ATPases: A Superfamily of Transport Proteins Operating From Escherichia Coli to Humans. Adv. Enzymol. Relat. Areas Mol. Biol..

[5] Paumi C.M., Chuk M., Snider J., Stagljar I., Michaelis S. (2009). ABC Transporters in Saccharomyces Cerevisiae and their Interactors: New Technology Advances the Biology of the ABCC (MRP) Subfamily. Microbiol. Mol. Biol. Rev. MMBR.

[6] Wannes D., Van Leeuwen T. (2014). The ABC Gene Family in Arthropods: Comparative Genomics and Role in Insecticide Transport and Resistance. Insect Biochem. Molec..

[7] Dean M., Hamon Y., Chimini G. (2001). The Human ATP-binding Cassette (ABC) Transporter Superfamily. J. Lipid Res..

[8] Labbé R., Caveney S., Donly C. (2011). Genetic Analysis of the Xenobiotic Resistance-Associated ABC Gene Subfamilies of the Lepidoptera. Insect Mol. Biol..

[9] Luo L., Sun Y., Wu Y. (2013). Abamectin Resistance in Drosophila is Related to Increased Expression of P-glycoprotein via the dEGFR and dAkt Pathways. Insect Biochem. Molec..

[10] Xu Z., Shi L., Peng J., Shen G., Wei P., Wu Q., He L. (2016). Analysis of the Relationship Between P-glycoprotein and Abamectin Resistance in Tetranychus Cinnabarinus (Boisduval). Pestic. Biochem. Phys..

[11] Gahan L.J., Pauchet Y., Vogel H., Heckel D.G. (2010). An ABC Transporter Mutation is Correlated with Insect Resistance to Bacillus thuringiensis Cry1Ac Toxin. PLoS Genet..

[12] Baxter S.W., Badenes-Pérez F.R., Morrison A., Vogel H., Crickmore N., Kain W., Wang P., Heckel D.G., Jiggins C.D. (2011). Parallel Evolution of Bacillus Thuringiensis Toxin Resistance in Lepidoptera. Genetics.

[13] Jin M., Tao J., Li Q., Cheng Y., Sun X., Wu K., Xiao Y. (2021). Genome Editing of the SfABCC2 Gene Confers Resistance to Cry1F Toxin from Bacillus Thuringiensis in Spodoptera Frugiperda. J. Integr. Agric..

[14] Tay W.T., Mahon R.J., Heckel D.G., Walsh T.K., Downes S., James W.J., Lee S., Reineke A., Williams A.K., Gordon K.H.J. (2015). Insect Resistance to Bacillus thuringiensis Toxin Cry2Ab is Conferred by Mutations in an ABC Transporter Subfamily a Protein. PLoS Genet..

[15] Pauchet Y., Bretschneider A., Augustin S., Heckel D.G. (2016). A P-Glycoprotein is Linked to Resistance to the Bacillus thuringiensis Cry3Aa Toxin in a Leaf Beetle. Toxins.

[16] Shengsong X., Bin S., Chaobao Z., Xingxu H., Yonglian Z. (2014). SgRNAcas9: A Software Package for Designing CRISPR sgRNA and Evaluating Potential Off-Target Cleavage Sites. PLoS ONE.

[17] Payton M.E., Greenstone M.H., Schenker N. (2003). Overlapping Confidence Intervals or Standard Error Intervals: What Do they Mean in Terms of Statistical Significance. J. Insect Sci..

[18] Shapira A., Livney Y.D., Broxterman H.J., Assaraf Y.G. (2011). Nanomedicine for Targeted Cancer Therapy: Towards the Overcoming of Drug Resistance. Drug Resist. Update..

[19] Chen Z., Shi T., Zhang L., Zhu P., Deng M., Huang C., Hu T., Jiang L., Li J. (2016). Mammalian Drug Efflux Transporters of the ATP Binding Cassette (ABC) Family in Multidrug Resistance: A Review of the Past Decade. Cancer Lett..

[20] Li Y., Zhang J., Chen D., Yang P., Jiang F., Wang X., Kang L. (2016). CRISPR/Cas9 in Locusts: Successful Establishment of an Olfactory Deficiency Line by Targeting the Mutagenesis of an Odorant Receptor Co-Receptor (Orco). Insect Biochem. Molec..

[21] Huang Y., Chen Y., Zeng B., Wang Y., James A.A., Gurr G.M., Yang G., Lin X., Huang Y., You M. (2016). CRISPR/Cas9 Mediated Knockout of the abdominal-A Homeotic Gene in the Global Pest, Diamondback Moth (*Plutella Xylostella*). Insect Biochem. Mol. Biol..

[22] Wu K., Shirk P.D., Taylor C.E., Furlong R.B., Shirk B.D., Pinheiro D.H., Siegfried B.D. (2018). CRISPR/Cas9 Mediated Knockout of the abdominal-A Homeotic Gene in Fall Armyworm Moth (*Spodoptera Frugiperda*). PLoS ONE.

[23] Zhu G., Xu J., Cui Z., Dong X., Ye Z., Niu D., Huang Y., Dong S. (2016). Functional Characterization of SlitPBP3 in *Spodoptera Litura* by CRISPR/Cas9 Mediated Genome Editing. Insect Biochem. Mol. Biol..

[24] Wei W., Huhu X., Bhaskar R., Junbiao D., Yungen M., Guanjun G. (2014). Heritable Genome Editing with CRISPR/Cas9 in the Silkworm, *Bombyx Mori*. PLoS ONE.

[25] Buss D.S., Mccaffery A.R., Callaghan A. (2002). Evidence for P-Glycoprotein Modification of Insecticide Toxicity in Mosquitoes of the *Culex Pipiens* Complex. Med. Vet. Entomol..

[26] Srinivas R., Udikeri S.S., Jayalakshmi S.K., Sreeramulu K. (2004). Identification of Factors Responsible for Insecticide Resistance in *Helicoverpa Armigera*. Comp. Biochem. Physiol. Part C.

[27] Buss D.S., Callaghan A. (2007). Interaction of Pesticides with P-Glycoprotein and Other ABC Proteins: A Survey of the Possible Importance to Insecticide, Herbicide and Fungicide Resistance. Pestic. Biochem. Phys..

[28] Aurade R.M., Jayalakshmi S.K., Sreeramulu K. (2010). P-Glycoprotein ATPase from the Resistant Pest, *Helicoverpa Armigera*: Purification, Characterization and Effect of Various Insecticides on its Transport Function. Biochim. Biophys. Acta.

[29] Zuo Y.Y., Huang J.L., Wang J., Feng Y., Han T.T., Wu Y.D., Yang Y.H. (2018). Knockout of a P-glycoprotein Gene Increases Susceptibility to Abamectin and Emamectin Benzoate in *Spodoptera Exigua*. Insect Mol. Biol..

[30] Yingchuan P., Jun Z., Yang S., Peng W., Yanyue H., Guanghua L., Wenjing Q., Shuijin H. (2021). Insights into Chlorantraniliprole Resistance of *Chilo Suppressalis*: Expression Profiles of ATP-binding Cassette Transporter Genes in Strains Ranging from Low- to High-Level Resistance. J. Asia-Pac. Entomol..

[31] Hawthorne D.J., Dively G.P. (2011). Killing them with Kindness? In-Hive Medications May Inhibit Xenobiotic Efflux Transporters and Endanger Honey Bees. PLoS ONE.

[32] Simmons J., D’Souza O., Rheault M., Donly C. (2013). Multidrug Resistance Protein Gene Expression in *T Richoplusia Ni* Caterpillars. Insect Mol. Biol..

[33] Tian L., Yang J., Hou W., Xu B., Xie W., Wang S., Zhang Y., Zhou X., Wu Q. (2013). Molecular Cloning and Characterization of a P-Glycoprotein from the Diamondback Moth, *Plutella Xylostella* (Lepidoptera: Plutellidae). Int. J. Mech. Sci..

[34] Kasorn T., Ping W. (2011). Differential alteration of two aminopeptidases N associated with resistance to *Bacillus thuringiensis* toxin Cry1Ac in cabbage looper. Proc. Natl. Acad. Sci. USA..

[35] Zhou J., Guo Z., Kang S., Qin J., Gong L., Sun D., Guo L., Zhu L., Bai Y., Zhang Z. (2019). Reduced Expression of the P-Glycoprotein gene *PxABCB1* is Linked to Resistance to Bacillus Thuringiensis Cry1Ac Toxin in *Plutella Xylostella* (L.). Pest Manag. Sci..

